# Inhibition of Hypoxia Inducible Factor Alpha and Astrocyte-Elevated Gene-1 Mediates Cryptotanshinone Exerted Antitumor Activity in Hypoxic PC-3 Cells

**DOI:** 10.1155/2012/390957

**Published:** 2012-11-29

**Authors:** Hyo-Jeong Lee, Deok-Beom Jung, Eun Jung Sohn, Hanna Hyun Kim, Moon Nyeo Park, Jae-Hwan Lew, Seok Geun Lee, Bonglee Kim, Sung-Hoon Kim

**Affiliations:** ^1^Cancer Preventive Material Development Research Center, College of Oriental Medicine, Kyung Hee University, 1 Hoegi-Dong, Dongdaemun-Gu, Seoul 130-701, Republic of Korea; ^2^BA/DDS Program, College of Arts and Science, New York University, New York, NY 10003, USA; ^3^Graduate School of East-West Medical Science, Kyung Hee University, Yongin 449-701, Republic of Korea

## Abstract

Although cryptotanshinone (CT) was known to exert antitumor activity in several cancers, its molecular mechanism under hypoxia still remains unclear. Here, the roles of AEG-1 and HIF-1**α** in CT-induced antitumor activity were investigated in hypoxic PC-3 cells. CT exerted cytotoxicity against prostate cancer cells and suppressed HIF-1**α** accumulation and AEG-1 expression in hypoxic PC-3 cells. Also, AEG-1 was overexpressed in prostate cancer cells. Interestingly, HIF-1**α** siRNA transfection enhanced the cleavages of caspase-9,3, and PAPR and decreased expression of Bcl-2 and AEG1 induced by CT in hypoxic PC-3 cells. Of note, DMOG enhanced the stability of AEG-1 and HIF-1**α** during hypoxia. Additionally, CT significantly reduced cellular level of VEGF in PC-3 cells and disturbed tube formation of HUVECs. Consistently, ChIP assay revealed that CT inhibited the binding of HIF-1**α** to VEGF promoter. Furthermore, CT at 10 mg/kg suppressed the growth of PC-3 cells in BALB/c athymic nude mice by 46.4% compared to untreated control. Consistently, immunohistochemistry revealed decreased expression of Ki-67, CD34, VEGF, carbonic anhydrase IX, and AEG-1 indices in CT-treated group compared to untreated control. Overall, our findings suggest that CT exerts antitumor activity via inhibition of HIF-1**α**, AEG1, and VEGF as a potent chemotherapeutic agent.

## 1. Introduction

Prostate cancer is one of the most common causes of cancer in men [1, 2]. Androgen ablation therapy and radical prostatectomy are the most common methods at the initial stages of this cancer. However, at the final stage, it develops into androgen-independent prostate cancer with aggravating and metastatic properties [3–5]. Most androgen-independent prostate cancers are easily hypoxic to show resistance to radiotherapy and chemotherapy [6]. Hypoxia inducible factor 1 alpha (HIF-1*α*) is involved in cancer angiogenesis, proliferation, erythropoiesis, and androgen resistance in prostate cancers [7–10]. Also, HIF-1*α* regulates prostate specific agent (PSA) expression via crosstalk with androgen receptor (AR) [11, 12], activates the transcription of the promoter of vascular endothelial growth factor (VEGF) as its downstream gene [13, 14], and promotes hypoxia-mediated expression of multidrug resistance 1 (MDR1) by binding to the hypoxia response element in the MDR1 promoter [15].

Astrocyte-elevated gene-1 (AEG-1) overexpressed in human brain and prostate cancers [16–18] promotes angiogenesis and metastasis [17, 18]. Recent studies show that AEG-1 is induced by hypoxia and glucose deprivation in glioblastoma [19], activates angiopoietin-1 (Ang1), matrix metalloproteinase (MMP)-2, and HIF-1 [20, 21], and plays a critical role in hepatocellular carcinoma progression as a target of microRNA-375 [22]. In addition, AEG-1 which is a downstream target of Ha-Ras enhances proliferation through downregulation of FOXO1 and inhibits apoptosis via activation of PI3K-Akt signaling [23, 24].

Recently, natural compounds are on the spotlight with low toxicity and chemotherapeutic properties [25–27]. Cryptotanshinone (CT), an abietane-diterpene derivative isolated from *Salvia miltiorrhiza* Bunge [28, 29], was known to have anti-proliferative [30, 31] and apoptotic [32, 33] activities in various cancer cells. Nevertheless, the antitumor mechanism of CT in association with HIF-1*α* and AEG-1 signaling in prostate cancers under hypoxia still remains unclear. Thus, in the present study, the molecular mechanism that CT suppresses hypoxia-induced AEG-1 and HIF-1*α* activation was investigated in androgen-independent prostate cancer PC-3 cells under hypoxia.

## 2. Materials and Methods

### 2.1. Compound

**Figure 1:**
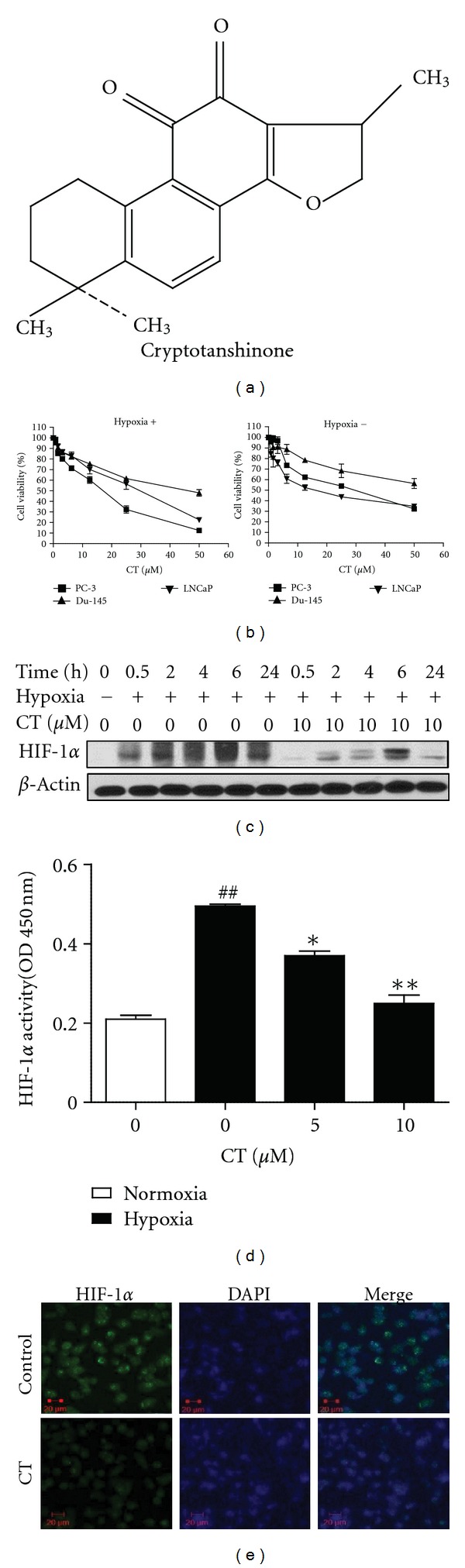
Effect of cryptotanshinone (CT) on hypoxia-induced HIF-1*α* activation in PC-3 cells. (a) Chemical structure of CT. (b) Effect of CT on the viability of prostate cancer cells under normoxia and hypoxia. (c) Effect of HIF-1*α* accumulation in hypoxic PC-3 cells. Cells were treated with CT (10 *μ*M) for 0.5, 2, 4, 6, and 24 h under hypoxia and Western blotting was performed. (d) Effect of CT on HIF-1*α* transcriptional activity in hypoxic PC-3 cells by ELISA. Cells were treated with or without CT (5 or 10 *μ*M) under normoxia or hypoxia for 6 h and HIF-1*α* transcriptional activity was evaluated by TransAM HIF-1 transcriptional factor assay kit. Data represent means ± S.D. ^##^ *P* < 0.01 versus normoxia control. *P < 0.05 or **P < 0,01 versus hypoxia control. (e) Effect of CT on HIF-1*α* nuclear translocation in hypoxic PC-3 cells. Cells treated with or without CT (10 *μ*M) under hypoxia for 6 h were fixed with 10% formalin for immunocytochemistry fluorescence staining. Green color was detected for HIF-1*α* in PC-3 cells, while nuclei were counterstained with blue color using DAPI.

Cryptotanshinone IIA (Figure 1(a)) purchased from Sigma was dissolved in dimethyl sulfoxide (DMSO) as a 10 mM stock solution for next experimental use.

### 2.2. Cell Culture and Hypoxia Treatment

PC-3 cells, DU145 (human androgen-independent prostate cancer), LNCaP (androgen-dependent prostate cancer), and RWPE-1 (noncancerous human prostatic epithelial cell line) cells were purchased from American Type Culture Collection (ATCC, Rockville, MD, USA). PC-3 and DU145 cells were maintained in RPMI 1640 (Welgene, Daegu, Korea) supplemented with 10% fetal bovine serum (FBS) and 1% antibiotic-antimycotic solution (Welgene, Daegu, Korea). LNCaP cells were maintained in RPMI 1640 medium supplemented with 10% fetal bovine serum (FBS), 0.45% D-glucose, 10 mM N-(2-hydoxy-ethyl) piperazine-N′-ethanesulfonic acid (HEPES) (Invitrogen, Carlsbad, CA, USA), and 1 mM sodium pyruvate (Invitrogen). RWPE-1 cells were grown in K-SFM containing 50 *μ*g/mL bovine pituitary extract, 5 ng/mL epidermal growth factor, and 1% antibiotic/antimycotic solution (Life Technologies, Inc., Grand Island, NY, USA). Human umbilical vein endothelial cells (HUVECs) were isolated from fresh human umbilical cord vein by collagenase treatment as previously described [34], which was approved by Kyung Hee hospital IRM committee (IRB No: KMC IRB0910-02). The cells were cultured in M199 supplemented with 20% heat-inactivated FBS, 3 ng/mL basic fibroblast growth factor (bFGF) (R&D Systems, Minneapolis, MN, USA), 5 units/mL heparin, and 100 units/mL antibiotic-antimycotic solution (Invitrogen) in 0.1% gelatin-coated flasks. HUVECs were used within passage three to seven. For hypoxia induction, cultures were incubated in a hypoxia chamber (Forma Anaerobic system, Asheville, NC, USA) with a gas mixture of 94% N_2_/5% CO_2_/1%O_2_.

### 2.3. Proliferation Assay

Proliferation assay was performed by using a bromodeoxyuridine (BrdU) assay kit (Roche Molecular Biochemicals, Mannheim, Germany) according to the manufacturer's instructions. PC-3 cells were transfected with control siRNA vector, siRNA HIF-1*α*, and siRNA AEG-1 (Santacruz biotechnology, Santacruz, CA, USA) at 50 nM by using INTERFERin siRNA transfection reagent (Polyplus-transfection Inc., New York, NY, USA). After 24 h transfection, PC-3 cells were incubated in hypoxia for an additional 24. Then BrdU was added to each well and incubated for 4 h to incorporate the pyrimidine analogue BrdU into the DNA of the cells. The cells were fixed by Fix dent solution and incubated with anti-BrdU antibody for 90 min. The immune complexes were detected by adding the substrate reaction to each well for 15 min. The reaction was stopped by adding 1 M H_2_SO_4_ and immediately quantified using a microplate reader (Tecan Group Ltd, Grödig, Austria) at 450 nm with a reference of 690 nm.

### 2.4. HIF-1 Alpha Transcription Activity Assay

HIF-1alpha transcription activity was analyzed by using TransAM HIF-1 transcription factor assay kit (Active Motif, Carlsbad, CA, USA) according to the instructions. Briefly, nuclear extracts were added onto 96-well microplate coated with oligonucleotides containing hypoxia response element (HRE) (5′-TACGTGCT-3′) from the erythropoietin (EPO) gene. HIF dimmers present in nuclear extracts bind with high specificity to response element and are subsequently detected with an antibody directed against HIF-1alpha. Addition of a secondary antibody conjugated to horseradish peroxidase (HRP) provides a sensitive colorimetric readout that is easily quantified by spectrophotometry. Values were expressed as optical density at 450 nm with a reference wavelength of 655 nm.

### 2.5. Tube Formation Assay

 Ice-cold Matrigel (BD Bioscience) was added to coat each well of 24-well plates and then polymerized by incubating for 1 h at 37°C. HUVECs (4 × 10^5^ cells/well) were treated with 500 *μ*l of culture supernatants from PC-3 cells treated either with or without CT (10 *μ*M) under hypoxia in the absence or presence of VEGF (20 ng/mL) (company). After 8 h incubation, the cells were fixed with 4% formaldehyde and randomly chosen fields were photographed under an Axiovert S 100 light microscope (Carl Zeiss, Weimar, Germany) at 100X magnification. Tube networks were quantified using NIH Scion image program.

### 2.6. Enzyme-Linked Immunosorbent Assay (ELISA) for VEGF

PC-3 cells were plated onto 60 mm dish at a density of 1 × 10^6^ cells/plate and incubated in the absence or presence of CT under normoxia or hypoxia for 24 h. VEGF level in the supernatant was measured by using human VEGF ELISA kit according to the manufacturer's protocol (Biosource International Inc., Camarillo, TX, USA).

### 2.7. Chromatin Immunoprecipitation (ChIP) Assay

PC-3 cells were plated onto 100 mm dishes at a density of 1.5 × 10^6^ cells/dish and exposed to CT for 4 h under normoxia or hypoxia. For ChIP, the EZ-zyme chromatin prep kit (Millipore, Billerica, MA, USA) was used following the manufacture's protocol. Briefly, PC-3 cells were plated onto 100 mm dishes at a density of 1.5 × 10^6^ cells/dish and exposed to CT for 4 h under normoxia or hypoxia. After incubation, the cells were fixed with 1% formaldehyde and incubated at room temperature for 10 minutes. After incubation, 10X glycine was added to dish and incubated for 5 minutes. Ez-zyme enzymatic cocktail was used to cleave the DNA (by following instruction manual). Protein-DNA complexes were immunoprecipitated with anti-HIF-1alpha antibody. The DNA-protein immunocomplexes were collected with protein A/G agarose beads, washed, and eluted with freshly prepared elution buffer (1%SDS, 0.1 M NaHCO_3_) with rotation at room temperature. The mixture was further incubated with 5 M sodium chloride at 65°C for 4 h to reverse crosslink DNA-protein complexes. Protein K (10 mg/mL) was added to the samples and incubated for 1 h at 45°C. DNA samples were then purified with phenol/chloroform, precipitated with ethanol, and resuspended in TE buffer. PCR reaction was performed to amplify VEGF promoter using ChIP primers (sense 5′-AGACTCCACAGTGCATACGTG-3′ and antisense 5′-AGTGTGTCCCTCTGACAATG-3′) and the effect of CT on the binding of HIF-1alpha to VEGF promoter was evaluated.

### 2.8. siRNA Transfection

PC-3 cells were transiently transfected with scramble, AEG-1 or HIF-1alpha siRNA (Santacruz biotechnology, Santacruz, CA, USA) at 50 nM by using INTERFERin siRNA transfection reagent (Polyplus-transfection Inc., New York, NY, USA). After incubation for 24 h, the cells were maintained for 24 h under hypoxia.

### 2.9. Western Blotting

Cells were lysed in radioimmunoprecipitation assay (RIPA) buffer (50 mM Tris, 300 mM NaCl, 5 mM ethylenediaminetetraacetic acid (EDTA), and 0.5% Triton X-100) containing protease inhibitors (10 *μ*g/mL aprotinin, 10 *μ*g/mL leupeptin, 10 mM iodoacetamide, 1% phenylmethylsulfonyl fluoride (PMSF), and 10 *μ*g/mL pepstain A) and phosphatase inhibitors (1 mM NaF and 1 mM Na_3_VO_4_). The protein samples were quantified by using RC DC protein assay kit II (Bio-rad, Hercules, CA, USA), separated on 6 to 10% SDS-polyacrylamide gels, and transferred to nitrocellulose membranes. The membranes were blocked with 5% nonfat milk and incubated with primary antibodies for HIF-1*α* (Becton Dickinson, Bedford, MA, USA), VEGF (Santa Cruz Biotechnology, Santa Cruz, CA, USA), AEG-1 (AbChem, USA), phosphorylated AKT, AKT (Cell signaling, USA), PARP (Santa Cruz Biotechnology, Santa Cruz, CA, USA), cleaved casepase-3 (Cell signaling, USA), cleaved caspase-9 (Cell signaling, USA), Bcl-2 (Santa Cruz Biotechnology, Santa Cruz, CA, USA), and *β*-actin (Sigma-Aldrich, St.Louis, MO, USA), followed by incubation with a horseradish peroxidase (HRP-) conjugated secondary antibodies. Protein expression was visualized by using enhanced chemiluminescence (ECL) Western blotting detection reagent (GE Health Care Bio-Sciences, Piscataway, NJ, USA).

### 2.10. Immunofluorescence Assay

Cells treated with HIF-1 siRNA or 500 *μ*M dimethyloxalylglycine (DMOG) (Enzo Life Sciences Plymouth Meeting, PA, USA) were fixed with 4% paraformaldehyde (PFA) and blocked in 0.1% Triton X-100/5% BSA in PBS for 25 min at 4°C and incubated with anti-AEG-1 (Ab Chem) or anti-HIF-1*α* (Santa Cruz Biotechnology, Santa Cruz, CA) for overnight at 4°C. Then, antirabbit IgG (1 : 1000) fluorescein isothiocyanate (FITC)-conjugate or antimouse IgG (1 : 1000) texas red-conjugate (Abcam, Cambridge, UK) was used as a secondary antibody for 1 hour at room temperature. The immunostained cells were mounted with mounting medium containing DAPI (1.5 *μ*g/mL) (Vectashield, Vector Labs, Burliname, CA, USA) and visualized by using Olympus FLUOVIEW FV10i confocal microscope.

### 2.11. PC-3 Xenograft Model

The animal study was conducted under guidelines approved by Institutional Animal Care and Use Committee, Kyung Hee University (KHUASP(SE)-09029). One million PC-3 cells were mixed with Matrigel (50%, in 100 *μ*l; Becton Dickinson, Bedford, MA, USA) and subcutaneously injected into the right flank 6-week-old male BALB/c athymic nude mice (Central Lab. Animal, Inc. Seoul, Korea). Two groups are control and CT treated groups in all. Each group consists of five mice. Three days after PC-3 cell inoculation, water contained 2% Tween 80 was i.p. injected into the mice of control group every other day, whereas CT (10 mg/kg) dissolved in 2% Tween 80 was i.p. injected into the mice in CT-treated group. Tumors were measured twice a week with a caliper, and tumor volume was calculated as described [26]. At the end of study, tumors were dissected, weighed, and photographed. Each tumor was fixed in 10% phosphate-buffered formalin for histology and immunohistochemistry (IHC).

### 2.12. Immunohistochemistry

Immunohistochemistry was carried out as previously described [35]. Antibodies for IHC in this study were Ki-67 (Lab vision Corporation, Fremont, CA, USA), VEGF (Santa Cruz Biotechnology, Santa Cruz, CA, USA), CD34 (Abcam, Boston, MA, USA), CAIX (Abcam, Boston, MA, USA) and AEG-1 (Abcam, Boston, MA, USA). For semiquantitation, 10 representative ×200 power photomicrographs were taken with a camera, avoiding gross necrotic areas. The positively stained cells within each photomicrograph were counted. The counting of total stained cells was aided with the ImagePro+ image-processing program.

### 2.13. Statistical Analyses

All data were expressed as means ± SD. The statistically significant differences between control and CT-treated groups were calculated by ANOVA test followed by a post hoc analysis (Tukey *t* or Dunnettes multiple comparison test) using Prism software 5 (GraphPad Software, Inc., San Diego, CA, USA).

## 3. Results

### 3.1. CT Exerts Significant Cytotoxicity in Prostate Cancer Cells under Hypoxia and Inhibits Hypoxia-Induced HIF-1*α* and AEG-1 Expression in Hypoxic PC-3 Cells

**Figure 2:**
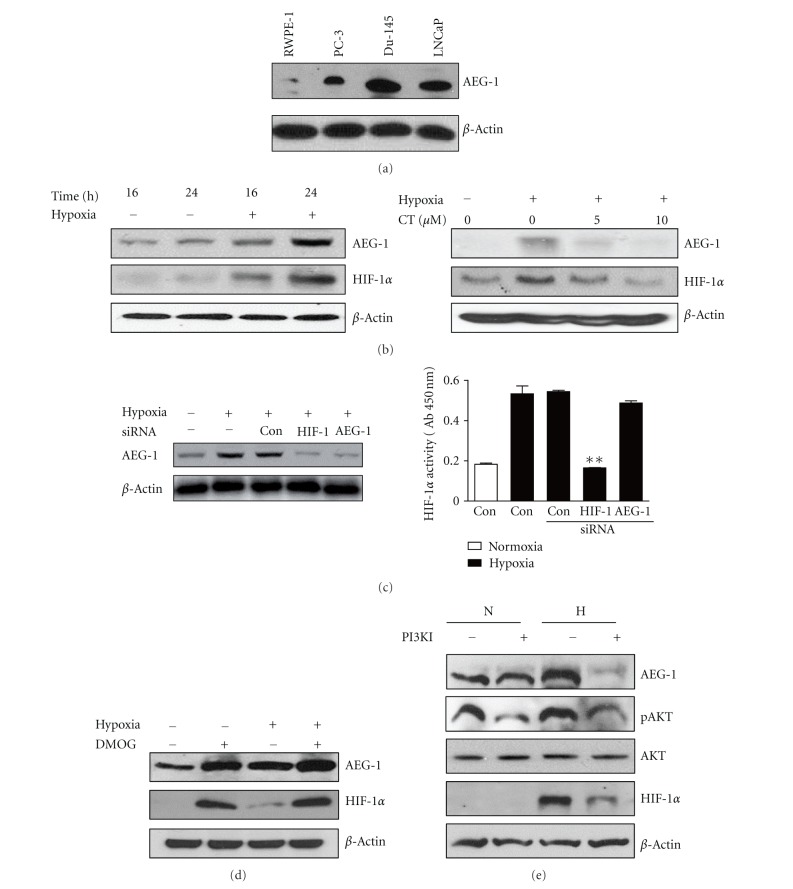
CT attenuates the expression of AEG-1 and HIF-1*α* in hypoxic PC-3 cells. (a) Expression of AEG-1 in various prostate cancer cells and normal prostate cells under hypoxia. Cell lysates were prepared and subjected to Western blotting. (b) Effect of hypoxia (left panel) or CT (right panel) on the expression of AEG-1 and HIF-1*α* in hypoxic PC-3 cells. (c) Effect of AEG-1 or HIF-1*α* siRNA transfection on AEG-1 expression by Western blotting (left panel) or HIF-1*α* activity by ELISA (right panel). (d) Effect of DMOG on the expression of AEG-1 and HIF-1*α* in hypoxic PC-3 cells. (e) Effect of PI3K inhibitor wortmannin (20 *μ*M) on AEG-1 or HIF-1*α* expression under normoxia and hypoxia by Western blotting.

 CT showed significant cytotoxicity against prostate cancer cells such as DU145, PC-3, and LNCaP cells under hypoxia more than under normoxia, which implies the potential of cryptotanshinone in resistant cancer cells, given that HIF-1*α* promotes the resistance to cancer cells [36] as shown in Figure 2(b). HIF-1*α* is an important factor that plays a role in cancer progression [37]. In the current study, CT exerted cytotoxicity against prostate cancer cells such as LNCaP, PC-3, and DU-145 cells under hypoxia (Figure 1(b)) and HIF-1*α* was dramatically induced under hypoxia, which was peaked at 6 h in PC-3 cells (Figure 1(c)). To investigate the effect of CT on hypoxia-induced HIF-1*α* accumulation, the cells were treated with CT under hypoxia and Western blotting was performed. CT significantly attenuated the protein expression of HIF-1*α* in a time- and concentration-dependent manners (Figure 1(c)). HIF-1*α* transcriptional activity assay was conducted at the time point (6 h) when HIF-1*α* expression was at the peak under hypoxia. CT effectively inhibited the HIF-1*α* transcriptional activity in a concentration-dependent manner (Figure 1(d)) by ELISA. Furthermore, immunocytochemical fluorescence staining revealed that CT attenuated nuclear translocation of HIF-1*α* in hypoxic PC-3 cells compared to untreated control (Figure 1(e)). 

### 3.2. Hypoxia Activates the Expression of AEG-1 in PC-3 Cells

AEG-1 was expressed in prostate cancer cells including DU145, LNCaP, and PC-3 cells. AEG-1 expression is significantly lower in RWPE-1 cells than in cancer cells (Figure 2(a)). To examine whether hypoxia affected the expression of HIF-1*α* and AEG-1 in PC-3 cells, Western blotting was carried out. Cells were cultured under normoxia or hypoxia for 16 h or 24 h. As shown in Figure 2(b) (left panel), HIF-1*α* and AEG-1 expressions were dramatically activated under hypoxia. Interestingly, treatment with 5 and 10 *μ*M of CT in hypoxia reduced the HIF-1*α* and AEG-1 expressions (Figure 2(b) right panel).

### 3.3. HIF-1*α* Regulates AEG-1 and PI3K Mediates Activation of HIF-1*α* and AEG-1 under Hypoxia

**Figure 3:**
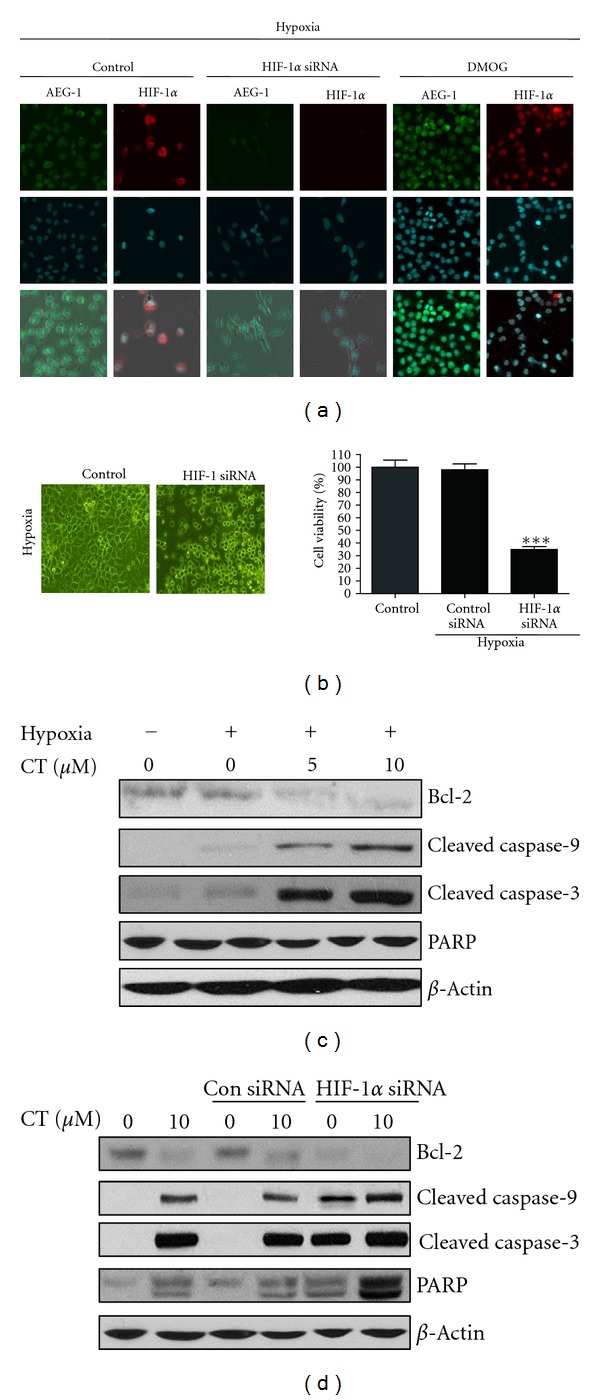
HIF-1*α* siRNA transfection attenuates AEG-1 expression and the viability of PC-3 cells under hypoxia, but DMOG enhances the expression of HIF-1*α* and AEG-1 in hypoxic PC-3 cells. (a) Effect of HIF-1*α* siRNA transfection or DMOG on HIF-1*α* stability and AEG-1 expression in hypoxic PC-3 cells. Cells were transfected with either control siRNA vector, HIF-1*α* siRNA, or AEG-1 siRNA and after 24 h were incubated in hypoxia for an additional 24 h. For DMOG treatment, Cells treated with DMOG for 24 hours under hypoxia were fixed with 10% formalin for immunocytochemistry fluorescence staining. Detection of HIF-1*α* (red) and AEG-1 (green) in PC-3 cells. Nuclei (blue) were counterstained using DAPI. (b) Effect of HIF-1*α* siRNA transfection on the viability of hypoxic PC-3 cells. Cells transfected with either non-target siRNA, HIF-1*α* siRNA were incubated under hypoxia for 48 h and the viability of the cells was evaluated by using BrdU assay. (c) Effect of cryptotanshinone on cell death in PC-3 cells. Cells were treated with cryptotanshinone (5, 10 *μ*M) for 48 h. (d) HIF-1*α* inhibition is necessary for CK-induced apoptotic cell death in PC-3 cells. PC-3 cells were transfected with either siRNA control or siRNA HIF-1*α* for 24 h and exposed to 10 *μ*M cryptotanshinone for 48 h.

HIF-1*α*, a heterodimeric transcription factor responding to hypoxia, is closely associated with numerous genes such as AEG-1 and PI3K [10]. Thus, the relationship between AEG-1 and HIF-1*α* was analyzed after transient transfection with HIF-1*α*, AEG-1, and control siRNA vector in hypoxic PC-3 cells. Here we found that HIF-1*α* was required for AEG-1 induction under hypoxia, since HIF-1*α* siRNA transfection suppressed the expression of AEG-1under hypoxia (Figure 2(c) left panel), while AEG-1 siRNA transfection did not block HIF-1*α* transcription activity in hypoxic PC-3 cells (Figure 2(c) right panel). Furthermore, to further confirm that HIF-1*α* regulated AEG-1 induction, DMOG as prolyl hydroxylase inhibitor was used in Western blotting. As shown in Figure 2(d), DMOG activated the expression of HIF-1*α* and AEG-1 in PC-3 cells under normoxia and hypoxia. Consistently, as shown in Figure 3(a), immunocytochemical fluorescence staining showed that HIF-1*α* siRNA transfection suppressed HIF-1*α* stabilization and AEG-1 induction, while DMOG enhanced them in hypoxic PC-3 cells.

There are some pieces of evidence that PI3K-Akt signaling is closely associated with AEG-1 under normoxia [23, 38]. Thus, the role of the PI3K pathway was comparatively investigated in AEG-1 induction during normoxia and hypoxia. PI3K inhibitor wortmannin (20 *μ*M) suppressed AEG-1and HIF-1*α* induction in PC-3 cells under hypoxia, while it did not affect AEG-1 or HIF-1*α* during normoxia (Figure 2(e)), indicating that AEG-1 is induced in either HIF-1*α* or PI3K-dependent pathway in hypoxic PC-3 cells.

### 3.4. CT Induces Apoptosis via Inhibition of HIF-1*α* under Hypoxia

To confirm whether absence of HIF-1*α* in hypoxic cancer cells can affect cell proliferation, the cells were transfected with HIF-1*α* siRNA. As expected, we found that HIF-1*α* siRNA transfection inhibited cell proliferation following hypoxia (Figure 3(b)). Other group's data were already reported that absence of HIF-1*α* inhibited cell proliferation and induced apoptosis in cancer cells. To confirm whether CT-induced apoptotic cell death by inhibition of HIF-1*α*, the effect of HIF-1*α* siRNA transfection on the CT induced apoptosis was elucidated in hypoxic PC-3 cells. As shown in Figure 3(c), CT induced the cleavages of caspase-9,3 and PAPR as well as attenuated the expression of Bcl-2 in hypoxic PC-3 cells. Furthermore, HIF-1*α* siRNA transfection enhanced the apoptosis induced by CT in hypoxic PC-3 cells as shown in Figure 3(d).

### 3.5. CT Exerts Antiangiogenic Activity via Inhibition of HIF-1*α* under Hypoxia

**Figure 4:**
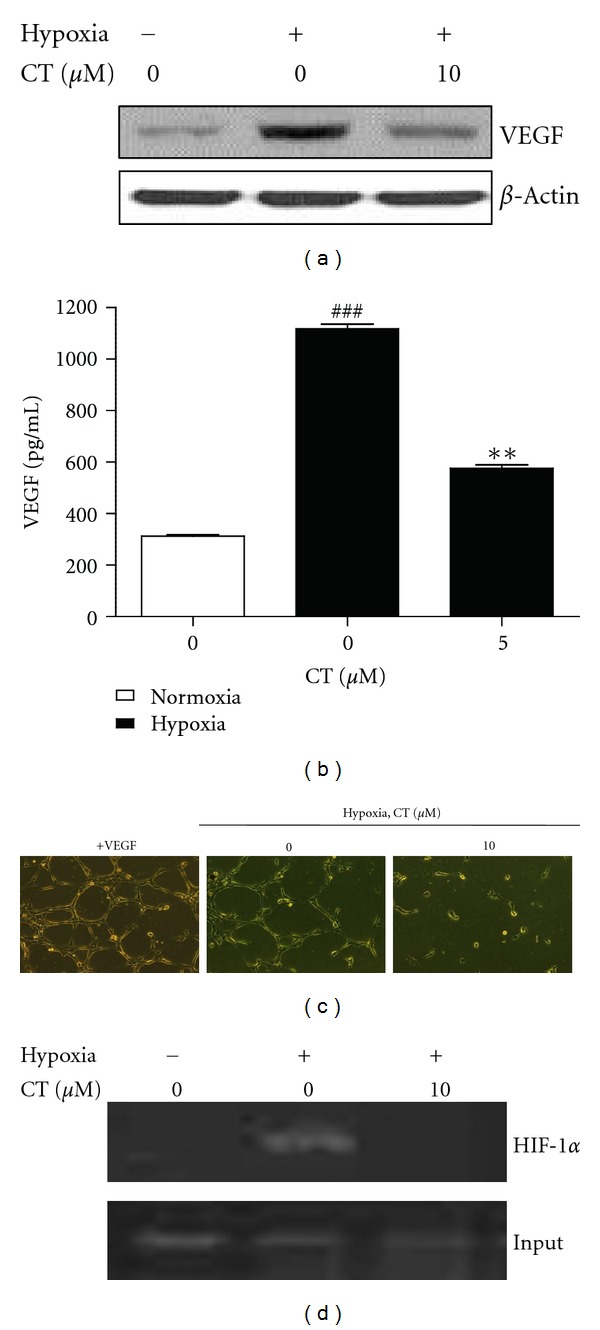
CT exerts antiangiogenic activity via inhibition of HIF-1*α* under hypoxia. (a) Effect of CT on the protein expression of VEGF in hypoxic PC-3 cells. Cell lysates were prepared and subjected to Western blotting to determine VEGF expression. (b) Effect of CT on the secreted production of VEGF in hypoxic PC-3 cells. VEGF levels in the culture supernatants were measured by using a Quantikine VEGF ELISA kit. Data represent means ± S.D. ^###^ *P* < 0.001 versus normoxia control. **P < 0,01 versus hypoxia control. (c) Effect of CT on tube formation in HUVECs treated with supernatant of PC-3 culture media. HUVECs were treated with VEGF (20 ng/mL) or the supernatant from PC-3 culture media with or without CT under hypoxia for 24 h. Tube formation assay was performed using growth factor-reduced matrigel. Cells were fixed with Diff-Quick solution, photographed randomly under an Axiovert S 100 light microscope at ×100 magnification. (d) Effect of CT on the binding of HIF-1*α* to VEGF promoter. Cells were treated with or without CT (5 or 10 *μ*M) under hypoxia for 6 h. The immunoprecipitated DNA with HIF-1*α* antibody was amplified by PCR analysis for VEGF promoter using ChIP primers.

Since hypoxia is one of angiogenesis inducers [39], the antiangiogenic effect of CT was evaluated under hypoxia. VEGF, a downstream gene of HIF-1*α*, was evaluated at the cellular and protein levels by ELISA and Western blotting. VEGF was more secreted and expressed in PC-3 cells under hypoxia compared to normoxia control. In contrast, CT attenuated hypoxia-induced VEGF protein expression (Figure 4(a)) and reduced VEGF secretion (Figure 4(b)) in hypoxic PC-3 cells. To further confirm antiangiogenic effect of CT, *in vitro* tube-formation assay was performed in HUVECs. HUVECs were cultured with conditioned medium collected from PC-3 cells in the absence or presence of CT under hypoxia. Consistent with the effect of CT on VEGF, tube formation under hypoxia was disturbed by CT treatment in PC-3 cells compared to untreated hypoxia control (Figure 4(c)). To find out whether HIF-1*α* directly binds to VEGF promoter, chromatin immunoprecipitation (ChIP) assay was conducted. As shown in Figure 4(d), the binding activity of HIF-1*α* to the VEGF promoter was detected under hypoxia compared to normoxia control. Notably, CT treatment effectively suppressed the binding of HIF-1*α* to VEGF promoter in hypoxic PC-3 cells.

### 3.6. CT Inhibits Tumor Growth in PC-3 Xenograft Model

**Figure 5:**
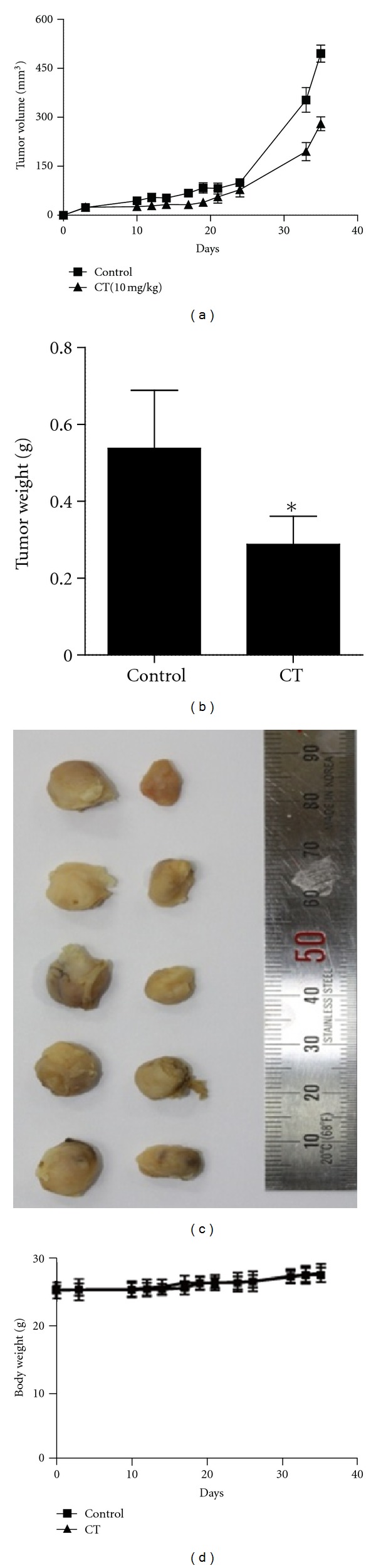
CT inhibits tumor growth in PC-3 xenograft model. (a) Effect of CT on the growth of PC-3 cells in BALB/c athymic nude mice. From 3 days after inoculation, CT (10 mg/kg body wt) was given by i.p. injection with 2% Tween-80 as vehicle every other days. Tumor growth was monitored twice a week. (b) Effect of CT on final tumor weight after sacrifice. Values represent means ± SD. *n* = 5. *P < 0.05 compared with untreated control. (c) Photographs of dissected tumors. (d) Effect of CT on body weights of mice at various time points. Values represent means ± SD. *n* = 5.

**Figure 6:**
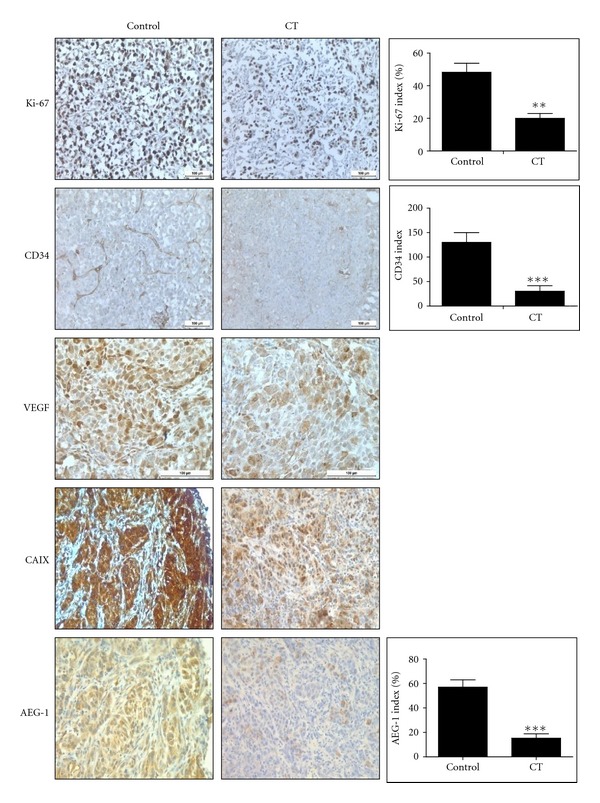
Effect of CT on the biomarkers of Ki-67, AEG-1, VEGF, CD34, and CAIX by immunohistochemistry. Immunohistochemistry was performed in tumor sections with antibodies of Ki-67 for proliferation, VEGF for angiogenesis, CD34 for microvessel density, CAIX for hypoxia, and AEG-1. The photographs were taken at ×200 magnification.

To confirm the *in vivo* antitumor efficacy of CT, tumor growth was monitored twice a week and immunohistochemistry was performed in PC-3 xenograft model. From 3 days after PC-3 cell inoculation, CT (10 mg/kg) was injected into the abdomen of BALB/c athymic nude mice every other day. As shown in Figures 5(a) and 5(c), CT treatment significantly retarded the growth of PC-3 cells in mice and decreased the final tumor weight by 46.4% without any side effects or body weight loss (Figure 5(d)). Also, immunohistochemistry for tumor sections revealed significant decreased expression of Ki-67 for proliferation, CD34 for microvessel density in the vascularized area of each tumor, and VEGF for angiogenesis (Figure 6). Carbonic anhydrase IX (CAIX) is frequently over-expressed in most human tumors as a biomarker for hypoxia [40, 41]. Given that AEG-1 correlated with hypoxia, AEG-1 was over-expressed in tumor sections similar to CAIX. In contrast, CT treatment suppressed the expression of AEG-1 and CAIX in tumor sections (Figure 6).

## 4. Discussion

The aim of this study was to elucidate the antitumor mechanism of CT isolated from Danshen *Salvia miltiorrhiza* in association with HIF-1*α* and AEG-1 signaling in PC-3 cells under hypoxia. HIF-1*α* is recognized as one of the most important microenvironmental factors that enable tumors to acquire an aggressive phenotype. HIF-1*α* becomes resistant to both chemotherapy and radiotherapy in cancers under hypoxia [42, 43]. CT exerted cytotoxicity against prostate cancer cells such as LNCaP, PC-3, and DU145 cells in a concentration-dependent manner and activated caspase 9/3 and PARP cleavage, implying mitochondrial-dependent apoptosis in PC-3 cells under normoxia or hypoxia. CT suppressed HIF-1*α* accumulation and transcriptional activity in PC-3 cells under hypoxia. This result implies that CT might attenuate hypoxia-mediated HIF-1*α* stability and transcription activity in hypoxic PC-3 cells.

AEG-1, since cloned in 2002, was known to have multibiological activities by regulating several signaling cascades such as NF-kappa B, PI3K/Akt, Wnt/ß catenin, H-Ras, c-Myc, and HIF1 alpha-related pathways in various cancer cells [23, 35, 44–46]. In the current study, AEG-1 was constitutively overexpressed in LNCaP, PC-3, and DU145 cells in normoxia. It was also upregulated along with HIF-1*α* in hypoxic PC-3 cells. In siRNA transfection assay to elucidate the relationship between HIF-1*α* and AEG-1, HIF-1*α* siRNA transfection blocked AEG-1 expression, while AEG-1 siRNA transfection did not affect HIF-1*α* activity in hypoxic PC-3 cells. This result strongly indicates that HIF-1*α* might regulate AEG-1 as an upstream gene under hypoxia.

Prolyl hydroxylase (PHD) plays an important role in the degradation of HIF-1*α* through the ubiquitin-proteasome system by hydroxylation in the presence of molecular oxygen [43, 46]. Consistently, PHD inhibitor, DMOG, enhanced the stability of HIF-1*α* and AEG-1 expression in hypoxic PC-3 cells under hypoxia by Western blotting and immunofluorescence assay, implying the critical role of PHD in regulation of HIF-1*α* and AEG-1 under hypoxia.

PI3K pathway is involved in AEG-1-mediated survival pathway and the transformed phenotype [23] and also linked to HIF-1*α* stabilization [47]. In the current study, PI3K inhibitor wortmannin (20 *μ*M) suppressed AEG-1and HIF-1*α* induction in PC-3 cells under hypoxia, while it did not affect AEG-1 or HIF-1*α* during normoxia (Figure 2(e)). These results suggest that PI3K pathway can regulate AEG-1 signaling in PC-3 cells under hypoxia. Similarly, Lee and his colleagues documented that PI3K signaling pathway augments binding of c-Myc to key E-box elements in the AEG-1 promoter, thereby regulating AEG-1 transcription, suggesting PI3K regulates AEG-1 as an upstream gene [23].

Hypoxia promotes decreased PHD enzymatic activity to induce HIF-1*α* accumulation and nuclear translocation by activating survival genes such as glycolysis (Glut1), VEGF, iron metabolism (transferrin), hypoxia pH control (CAIX), and hemoglobin synthesis (erythropoietin) [46]. Here CT significantly reduced cellular levels of VEGF, a downstream of HIF-1*α*. It also disturbed tube formation in human umbilical vein endothelial cells (HUVECs) maintained in conditioned medium of hypoxic PC-3 cells, suggesting the antiangiogenic activity of CT in hypoxic PC-3 cells. Furthermore, ChIP assay revealed that CT inhibited the binding of HIF-1*α* to VEGF promoter, demonstrating that CT inhibits HIF-1*α*-mediated angiogenesis by suppressing its binding to VEGF.

In addition, CT at 10 mg/kg suppressed the growth of PC-3 cells in BALB/c athymic nude mice by 46.4% compared to untreated control without weight loss or side effects. Furthermore, consistent with its *in vitro* efficacy, CT effectively decreased the expression of Ki-67 for proliferation biomarker, CD34 for microvessel density, VEGF for angiogenic biomarker, CAIX for hypoxia biomarker, and AEG-1 indices in PC-3 bearing nude mice compared to untreated control, indicating the antitumor effect of CT was exerted by inhibition of proliferation, angiogenesis, and hypoxia-related genes. Although CT was known to have antiproliferative [30], apoptotic [32, 33], and antiangiogenic activities in cancers in normoxia [48, 49], the antitumor activity of CT in hypoxic PC-3 cells was associated with PI3K, HIF-1*α* and AEG-1, signaling pathways in the current study.

In summary, CT suppressed HIF-1*α* accumulation and transcriptional activity and AEG-1 expression in hypoxic PC-3 cells. However, HIF-1*α* siRNA transfection blocked AEG-1 expression, while AEG-1 siRNA transfection did not affect HIF-1*α* activity in hypoxic PC-3 cells. Also, HIF PHD inhibitor, DMOG, enhanced the stability of HIF-1*α* and AEG-1 during hypoxia. In addition, CT also significantly reduced cellular levels of VEGF and disturbed tube formation in HUVECs maintained in conditioned medium of hypoxic PC-3 cells by inhibition of the binding of HIF-1*α* to VEGF promoter. Furthermore, CT at 10 mg/kg suppressed the growth of PC-3 cells in BALB/c athymic nude mice by 46.4% and effectively decreased the expression of Ki-67, CD34, VEGF, CAIX, and AEG-1 indices by immunohistochemistry compared to untreated control. Taken together, our findings suggest that CT exerts antitumor activity via inhibition of HIF-1*α*, AEG1, and VEGF as a potent chemotherapeutic agent.
