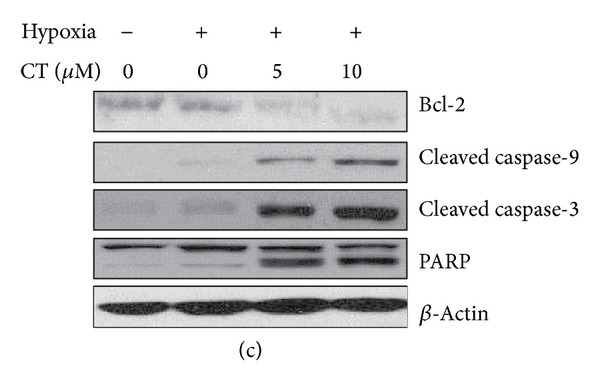# Erratum to “Inhibition of Hypoxia Inducible Factor Alpha and Astrocyte-Elevated Gene-1 Mediates Cryptotanshinone Exerted Antitumor Activity in Hypoxic PC-3 Cells”

**DOI:** 10.1155/2013/267352

**Published:** 2013-10-09

**Authors:** Hyo-Jeong Lee, Deok-Beom Jung, Eun Jung Sohn, Hanna Hyun Kim, Moon Nyeo Park, Jae-Hwan Lew, Seok-Geun Lee, Bonglee Kim, Sung-Hoon Kim

**Affiliations:** ^1^Cancer Preventive Material Development Research Center, College of Oriental Medicine, Kyung Hee University, 1 Hoegi-Dong, Dongdaemun-Gu, Seoul 130-701, Republic of Korea; ^2^BA/DDS Program, College of Arts and Science, New York University, New York, NY 10003, USA; ^3^Graduate School of East-West Medical Science, Kyung Hee University, Yongin 449-701, Republic of Korea

An error occurred during uploading Figure 3(c), and the following is the correct figure.

## Figures and Tables

**Figure 3:**